# Expression of Behavioral Phenotypes in Genetic and Environmental Mouse Models of Schizophrenia

**DOI:** 10.3389/fnbeh.2020.00029

**Published:** 2020-02-28

**Authors:** Razia Sultana, Charles C. Lee

**Affiliations:** Neural Systems Laboratory, Department of Comparative Biomedical Sciences, Louisiana State University School of Veterinary Medicine, Baton Rouge, LA, United States

**Keywords:** DISC1 (disrupted-in-schizophrenia 1), maternal separation (MS), NMDAR hypofunction, ketamine injections, schizophrenia-like psychoses, gene-environment (G-E) interaction

## Abstract

Schizophrenia is a neuropsychiatric disorder characterized by multifactorial etiology involving complex interactions among genetic and environmental factors. “Multiple-hit” models of the disorder can explain its variable incidence and prevalence in related individuals. Hence, there is a dire need to understand these interactions in the emergence of schizophrenia. To test these factors in the emergence of schizophrenia-like behaviors, we employed a genetic mouse model of the disorder (harboring the DISC1 mutation) along with various environmental insults, such as early life stress (maternal separation of pups) and/or pharmacological interventions (ketamine injections). When assessed on a battery of behavioral tests, we found that environmental interventions affect the severity of behavioral phenotypes in terms of increased negative behavior, as shown by reduced mobility in the forced swim and tail suspension tests, and changes to positive and cognitive symptoms, such as increased locomotion and disrupted PPI along with reduced working memory, respectively. Among the various interventions, the genetic mutation had the most profound effect on behavioral aberrations, followed by an environmental intervention by ketamine injections and ketamine-injected animals that were maternally separated during early postnatal days. We conclude that although environmental factors increased the prevalence of aberrant behavioral phenotypes, genetic background is still the predominant influence on phenotypic alterations in these mouse models of schizophrenia.

## Introduction

Schizophrenia is a neuropsychiatric disorder whose etiology encompasses the interaction of several genetic and environmental factors. Heritability of the disorder is as high as 80% (Sullivan et al., 2003), with considerable ecogenetic variation in the prevalence of the disease among related individuals (Ettinger et al., 2004). Such variation correlates with the degree of genetic relatedness of affected individuals; prevalence in first degree relatives (4%–8%), second-degree relatives (2%–3.5%), and children of affected individuals (one parent affected, 13.6%; and both the parents affected, 37%) is indicative of the genetic heritability of the disorder (Salleh, 2004). The most pronounced variations exist in twin studies with a concordance of 50% (Cardno and Gottesman, 2000), suggesting a multifactorial etiology for schizophrenia and related disorders beyond genetic predisposition.

Epidemiological studies of the disease show pronounced interactions between genetic and environmental factors, which can explain variable degrees of onset, prevalence, and severity of disorders in different individuals with a genetic predisposition for the disorder (Karl and Arnold, 2014). Among the environmental factors, maternal separation, early life stress, drug abuse, and season and place of birth are related to the clinical presentation of schizophrenia (Tsuang, 2000; Tsuang et al., 2001; Morgan and Fisher, 2006). Such an interplay of genetics and environment has given rise to “multiple-hit” models of schizophrenia and associated psychotic disorders, where both genes and environment are important factors for disease expression in humans, as well as in animal models of psychotic disorders (Bayer et al., 1999; Maynard et al., 2001; McGrath et al., 2003; Feigenson et al., 2014).

Several candidate genes have been associated with schizophrenia through genome-wide association (GWA) and single nucleotide polymorphisms (SNPs) studies (McClellan et al., 2007; Gejman et al., 2010). Among these, the *DISC1* (Disrupted in Schizophrenia 1) gene confers a 2% risk of schizophrenia in carriers (Callicott et al., 2005; Song et al., 2008; Williams et al., 2009). DISC1 is a scaffolding protein that interacts with several genes, such as NDE, NUDEL, PDE4, ATF4 and PCM etc. (Blackwood et al., 2001; Brandon et al., 2009; Porteous and Millar, 2009; Bradshaw and Porteous, 2012; Teng et al., 2018). In particular, its interaction with PDE4 in dendritic spines serves as a molecular brake to maintain levels of cAMP to restore synaptic connectivity in the PFC (Soares et al., 2011). Due to its synaptic localization with PDE4 and HCN channels it plays a vital role in maintaining working memory and other related behavioral phenotypes (Niwa et al., 2010; Gamo et al., 2013; Paspalas et al., 2013).

Several human and animal studies have further demonstrated the role of mutations in the *DISC1* gene that lead to differential disease phenotypes with variable prevalence (Gottesman and Shields, 1976; Munafò et al., 2005; Van Winkel et al., 2010; Uher, 2014). In humans, a focused study of a Scottish family with this mutation, 33.3% of individuals exhibited symptoms of schizophrenia, major depression (47%), adolescent misconduct (9.5%), bipolar and minor depression, respectively (4.7%; Hennah et al., 2006). Furthermore, a frameshift mutation of the *DISC1* gene in an American family was associated with schizophrenic and schizotypic affective disorders (Sachs et al., 2005; Zhang et al., 2006). In animal studies, such as in the 129S inbred strain of mouse (with a spontaneous, native truncation in the C-terminal of DISC1), L100 and Q31l mutations of the gene result in schizophrenia-like phenotypes (Clapcote and Roder, 2006; Clapcote et al., 2007; Niwa et al., 2010; Sultana et al., 2019). With its relative prevalence and concordant disease expression, DISC1 mutations are an important genetic factor in the etiology of schizophrenia pathogenesis.

Despite the advances in understanding the genetic and environmental factors involved in the etiology of schizophrenia and a plethora of molecular interactions of DISC1 protein at presynaptic, synaptic and/or postsynaptic sites (Hikida et al., 2012; Weng et al., 2018; Barnett et al., 2019) it remains unclear the degree to which the *DISC1* gene interacts with environmental stressors and how such interactions impact the disease presentation in affected individuals. Therefore to understand the behavioral impact of DISC1 interactions with environmental insults, in the present study, we utilized a mouse model of schizophrenia (Jones et al., 2011) to test the effects of an environmental stressor (maternal separation) and/or pharmacological intervention (ketamine; Table 1) on the severity of behavioral phenotypes in genetically predisposed animals (with *DISC1* mutation) compared with controls. We found that although environmental variables increased the number of animals exhibiting aberrant behaviors, the genetic composition of the animals was still the major driver in the expression of schizophrenia-related phenotypes.

**Table 1:** Description of all the intervention groups used.

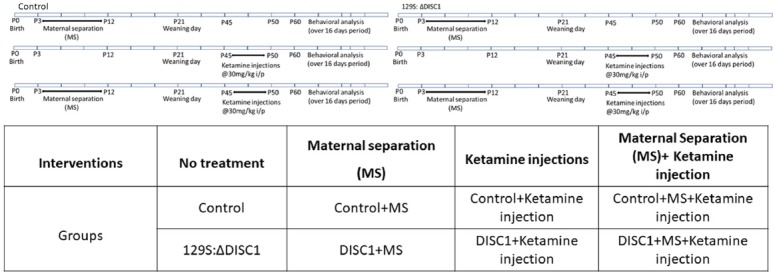

## Materials and Methods

### Animal Care and Housing

A total of 12 animals were used in each group: 129SvEv (129S:ΔDISC1) a DISC1 mutation model and C57BL/6J (control), and the intervention groups described below. Mice were obtained from the Jackson Laboratory (Bar Harbor, ME, USA). These animals were assessed on a battery of behavioral tests when 8 weeks old, with the least stressful tests performed first (Sultana et al., 2019). Initially, control and 129S:ΔDISC1 animals were characterized by behavior without any interventions. Animals were housed in a temperature and humidity-controlled room with a 12 h light/dark cycle with lights on at 7:00 am and food and water provided *ad libitum*. All the experiments were conducted according to NIH guidelines and were approved by the Institutional Animal Care and Use Committee (IACUC) of the Louisiana State University School of Veterinary Medicine.

### Animal Models and Interventions

#### Maternal Separation (MS; Early Life Environmental Stress)

Maternal separation of newborns, young children have shown a strong correlation with psychotic disorder precipitation in human subjects (Mäki et al., 2003; Paksarian et al., 2015). Maternal separation in mouse and rat pups, in particular, has been used to model and study schizophrenia (Lehmann et al., 2000; Fabricius et al., 2008). In this study, maternal separation of pups was performed with slight modifications to previously described procedures (Roceri et al., 2002; Ellenbroek and Riva, 2003). The pups were separated from dams for 4 h a day from postnatal day (PND) 3 to PND12 (critical period of brain development at these stages; Rice and Barone, 2000) daily from 10:00 a.m. to 2:00 p.m. and weaned at PND21. The animals were tested after PND60.

#### NMDAR Hypofunction (i.p. Ketamine Injection)

NMDAR hypofunction is a convergent molecular deficit found in instances of schizophrenia. To induce a pharmacologically targeted behavioral deficit, we used a previously established model of schizophrenia (Ogundele and Lee, 2018) To induce NMDAR hypofunction similar to molecular findings in schizophrenia, both 129S:ΔDISC1 and control animals were injected with a subanesthetic dose of ketamine (30 mg/kg) for 5 days from day 45–50, as described previously (Becker et al., 2003; Frohlich and Van Horn, 2014; Ogundele and Lee, 2018) and were tested behaviorally starting at 5–7 days following the last ketamine injection.

#### Maternal Separation (PND3–PND12) With i.p. Ketamine Injection During Adulthood

Both 129S:ΔDISC1 and control animals were separated maternally (from PND3-PND12; 4 h a day). In adulthood, they were injected with ketamine (i.p. 30 mg/kg), as described above. These animals were then tested under the behavioral test battery as follows.

#### Behavioral Test Battery

All behavioral experiments were performed by the same investigator during the late morning. The behavioral procedures have been described in detail in our previous work (Sultana et al., 2019). One set of experiments was performed per day over a 16 days period with resting days in between. Experiments were performed in the order as we have described in our prior study (Sultana et al., 2019). The following tests were included.

### Open Field Test for Thigmotaxis and Overall Activity

The total distance traveled in the apparatus was calculated and used as a measure of overall activity (Foshee et al., 1965). In addition, this test was also used as a measure of anxiety-like behavior in terms of thigmotaxis, i.e., time spent near the periphery of the chamber (Simon et al., 1994; Seibenhener and Wooten, 2015; Walz et al., 2016). Thus, we also determined the percent time the test animal spent at the periphery vs. center (Sultana et al., 2019) during the total 5 min test duration.

### Sociability and Novelty

As a measure of social interaction, sociability and social novelty were tested as previously described (Kaidanovich-Beilin et al., 2011). On the test day, animals were assessed for sociability, as defined by the percent time that the test animal spent socializing with stranger 1 (S1) i.e., (S1/S1+E) * 100. Social novelty was assessed as the percent time spent with stranger 2 (S2) as (S1/S1+S2) * 100.

#### Modified Porsolt Forced Swimming Test

As a metric of despair, we utilized the modified Porsolt forced swimming test, derived from the procedure of Can et al. (2012a,b). The camera (1080 HD, Logitech, Newark, CA, USA) was positioned with a side view of the beaker to record the leg movements of the animal. Scoring of the movements was done as previously described by Can et al. (2012a,b). Percent mobility time was calculated from a total 4 min testing period, following an initial 2 min acclimation period which was later excluded from calculations. Measurements included when the animal was actively struggling to escape from the water container, whereas the propelling movement was not considered in the mobility calculations.

#### Tail Suspension Test

This test was used as another metric of negative behavior, animals were suspended by a custom holder and percent mobility during suspension was assessed (Can et al., 2012b). The total test duration was 6 min, but the latter 4 min were analyzed to remove any bias involving acclimation.

#### Stress Calls

When interpreted contextually, in certain psychotic disorders like schizophrenia and schizotypic affective disorders, ultrasonic vocalization (USV) patterns can provide an indicator of the affective state of the animal (Knutson et al., 2002; Schwarting and Wöhr, 2012; Mun et al., 2015). Stress calls were recorded simultaneously to the tail-suspension test, as described previously. An AT125 bat call recorder (Binary Acoustics, Carlisle, PA, USA) and digital recording software SPECT’R (Binary Acoustics, Carlisle, PA, USA) was used. Calls were analyzed offline using SCAN’R software (Binary Acoustics, Carlisle, PA, USA). Calls above 30 kHz (typical of adult mice) with a minimum duration of 5 ms were considered and analyzed. The number of calls per 6 min period was calculated along with the mean and maximum frequency and the duration of each call. The entire 6 min period was analyzed since no difference was found when examining these data compared to the 4 min period post-acclimation.

#### Y-Maze Test

Working memory includes the ability to rapidly form a memory trace and the exclusion of old information from that which is currently valid. This task was used as a tool to assess schizophrenia-related cognitive impairment. Videos were recorded using a Logitech HD 1180 camera and later analyzed with ANY-maze (ANY-maze, Wood Dale, IL, USA). Percent entries into the correct arm were calculated using the formula described previously (Sultana et al., 2019).

#### Habituation to Acoustic Startle and Pre-pulse Inhibition (PPI)

As a measure of pre-attentive deficits, this test is also used to assess sensorimotor gating in human subjects with schizophrenia (Swerdlow et al., 2006). Following the protocol described by Valsamis and Schmid (2011), responses to acoustic startle stimuli were used to measure habituation and pre-pulse inhibition (PPI). The apparatus and protocols were followed as described in our prior study (Sultana et al., 2019). For stimulus delivery and recording of the startle signal, Audacity software 2.2.2 (Carnegie Mellon University, Pittsburgh, PA, USA) was used. The startle data were exported into Excel (Microsoft, Redmond, WA, USA) using Python. Further analysis was done using Excel followed by statistical analysis with GraphPad Prism 5 (LaJolla, CA, USA). The data were expressed as mean ± SEM.

### Statistical Analysis

ANOVA followed by Tukey’s *post hoc* test for multiple comparisons was used to determine significant differences among groups. All the data were expressed as mean ± SEM. A *p*-value < 0.05 was considered statistically significant. Statistical analysis was performed using GraphPad Prism 5 (GraphPad Software, La Jolla, CA, USA).

## Results

### Thigmotaxis, Anxiety-Related Behavior

**Figure 1:**
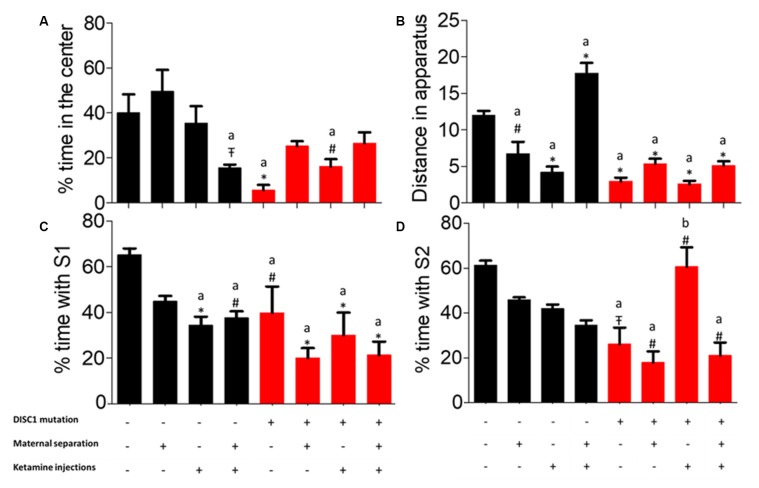
Three-chambered test for **(A)** percent time spent at the center of open field. **(B)** Total distance traveled throughout the apparatus. **(C)** Sociability. **(D)** Novelty. All data presented here are presented as mean ± SEM, where a* compared to control at *p* ≤ 0.001; a# as compared to control at *p* ≥ 0.05; a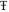 as compared to control at *p* ≤ 0.01 and b with above symbols show comparison of groups with DISC1 mutation animals.

As noted previously, time spent by animals along the walls of the open field provides an index of anxiety (Sultana et al., 2019). Here, we calculated the percent time that animal groups spent at the periphery. We found that control animals spent a significantly higher time in the center vs. control+MS+ketamine (*p* ≤ 0.05; see Figure 1A), while animals with the *DISC1* mutation showed a variable degree of time at the periphery differing from control animals (DISC1 alone at *p* ≤ 0.001 and DISC1+ketamine at *p* ≤ 0.01). Different interventions on the control background did not affect this behavior, except in the control+MS+ketamine group (at *p* ≤ 0.05; Figure 1A). The variable degree of anxiety-related behavior was intriguing, since not all the interventions on the DISC1 and/or control group background exhibited this behavior (37.5% of the population among all the groups tested here), compared to schizophrenia subjects, anxiety-related behavior is not the endophenotype, with a 38% prevalence of this behavior in human schizophrenia patients (Temmingh and Stein, 2015).

### Exploratory Behavior and Activity

While in the open field, the total distance covered by all groups was assessed. Control animals traveled a significantly greater distance as compared to other groups, i.e., DISC1 animals alone and with interventions (*p* ≤ 0.001; Figure 1B). Environmental interventions on the DISC1 background did not affect exploratory behavior when compared to the DISC1 without intervention animals. Moreover, control animals with maternal separation, ketamine injection showed significantly decreased exploration (*p* ≤ 0.01 and *p* ≤ 0.001, respectively). These results support the interpretation that all the environmental interventions affect animal behavior differently depending on the genetic background and their specific interactions.

### Sociability and Novelty

Control animals exhibited significantly higher time socializing compared with other groups, with the exception of maternally separated (MS) controls (where different groups differed at *p*-values control+ketamine at *p* ≤ 0.001, control+MS+ketamine at *p* ≤ 0.01, DISC1 at *p* ≤ 0.01, along with DISC1+MS, DISC1+ketamine, DISC1+MS+ketamine at *p* ≤ 0.001, respectively; see Figure 1C). The intervention groups did not exhibit an intragroup difference in percent time with S1 (stranger 1) when compared amongst themselves, as shown in Figure 1C. These results indicate that environmental interventions equally affect social withdrawal in all the models, with a higher degree of social isolation in genetic mutation animals (significant at *p* ≤ 0.01 in all *DISC1* background groups). However, environmental interventions on a DISC1 mutation background did not change the sociability behavior of these animals significantly.

Social novelty (i.e., percent time with S2) exhibited a different outcome (Figure 1D), with control animals differing from the DISC1 (*p* ≤ 0.05), maternally separated DISC1 (*p* ≤ 0.01), MS+DISC1 injected with ketamine at adulthood (*p* ≤ 0.01), and the DISC1+ ketamine injection animals exhibited an increased social novelty vs. DISC1 animals alone (*p* ≤ 0.01), suggesting a complex interplay in DISC1 animals with pharmacologically induced NMDAR hypofunction (using ketamine; Figure 1D). Additionally, other environmental interventions in DISC1 animals did not differ significantly from those with no interventions. Control animals did not show a significant difference when environmental interventions were imposed on this background, indicating that the genetic mutation in DISC1 animals influenced their behavior towards social novelty.

### Y Maze: As an Index of Working Memory

**Figure 2:**
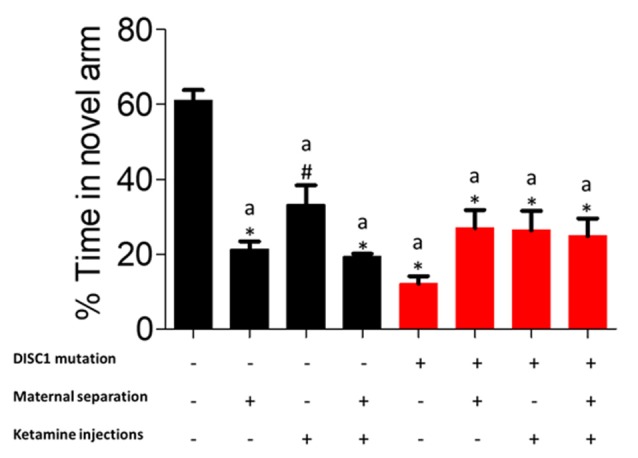
Y-maze task for working memory: showing percent time each group spent in the novel arm. All data presented here are presented as mean ± SEM, where a* as compared to control at *p* ≤ 0.001; a# as compared to control at *p* ≤ 0.05.

In this behavioral task, control animals exhibited significantly higher percent time in the novel /correct arm (previously blocked arm) as compared to DISC1 (DISC1 alone and with interventions at *p* ≤ 0.001) and other groups (control+MS and control+MS+ketamine at *p* ≤ 0.001 and control+MS at *p* ≤ 0.01; Figure 2). It is important to note that environmental interventions on a DISC1 mutation background did not significantly disrupt the results of the working memory task when compared to DISC1 animals without interventions. These results demonstrate that working memory is affected in all the control animal groups with various environmental interventions, exhibiting the effect of these stressors on the hippocampus (Malhotra et al., 1997; de Azeredo et al., 2017). Percent novel arm entries did not show any significant difference among control vs. intervention groups (data not shown).

### Porsolt Forced Swim Test (FST)

**Figure 3:**
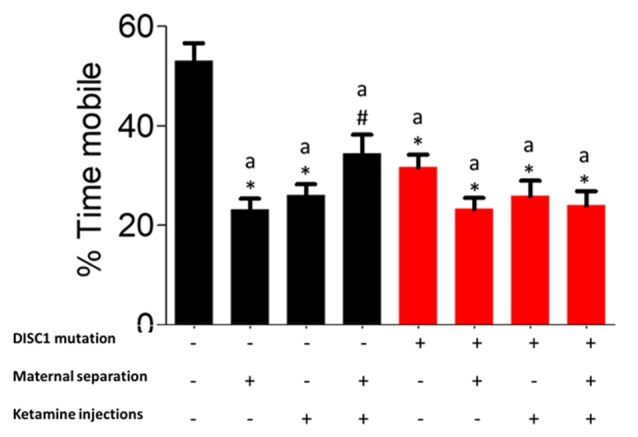
Forced Swim Test (FST) for depressive behavior: exhibiting the percent mobility time by all the groups. All the presented here are presented as mean ± SEM, where a* as compared to controls at *p* ≤ 0.001; a# as compared to controls at *p* ≤ 0.05.

Mobility during forced swim test (FST) can be used as a measure of the degree of despair in animal models of behavioral disorders (Can et al., 2012a). When test groups were compared for mobility timing in FST, we found a variation in the number of animals exhibiting depressive behavior within each group, whereas when statistically compared, there was a significant reduction in mobility timing (DISC1, DISC1+MS, DISC1+MS+ketamine and control+ketamine, control+MS+ketamine at *p* ≤ 0.001; and at *p* ≤ 0.01 for control+MS; as shown in Figure 3). The environmental factors over a DISC1 mutation background did not show a significant reduction in mobility time when compared to DISC1 mutation animals without intervention. Although maternal separation (Millstein and Holmes, 2007), ketamine injections, and combinations of both interventions affect the animals of each group to a variable degree, these groups still exhibit an overall depressed phenotype (Elk et al., 1986).

### Tail Suspension Test and Stress Calls

**Figure 4:**
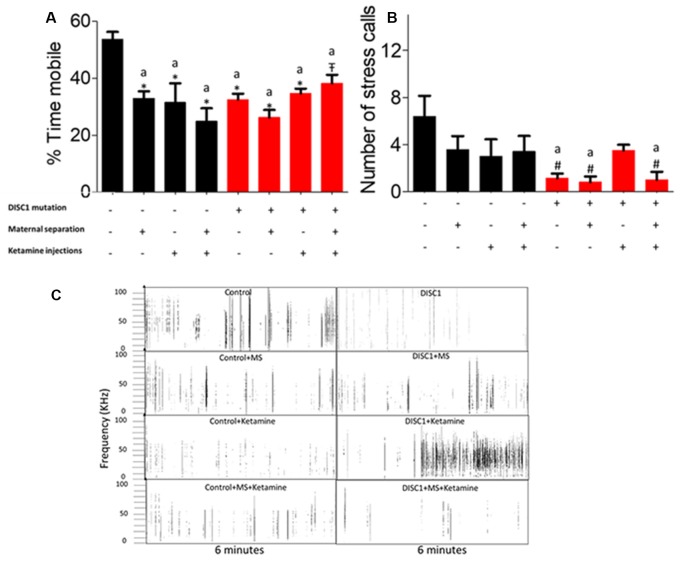
Tail Suspension Test (TST) for depressive behavior: **(A)** exhibiting the percent mobility time by all the groups. **(B)** Stress calls recorded/6 min period while under tail suspension. **(C)** Representative stress calls in all the groups; with y-axis showing frequency/call and x-axis showing duration (6 min). All data presented here are presented as mean ± SEM, where a* as compared to control at *p* ≤ 0.001; a# as compared to control at *p* ≤ 0.05. a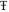 as compared to control at *p* ≤ 0.01 and *b* with above symbols shows comparison of groups with DISC1 mutation animals.

The tail suspension test was used as another measure of despair and resulted in a similar outcome as the FST. Control animals showed the highest mobility, which was significantly different from other groups (at *p* ≤ 0.001 for all the groups and *p* ≤ 0.01 for DISC1+MS+Ketamine vs. control; see Figure 4A). DISC1 mutation animals vs. DISC1 with other interventions did not show significant differences amongst themselves. Thus, various environmental stressors led to decreased mobility in the groups which was not significantly different from each other (Figure 4).

While the animals were suspended, we also recorded the USV emitted by these animals, as a measure of calls produced under stress. We found that *DISC1* mutation animals produced fewer calls compared to control animals (*p* ≤ 0.01; Figure 4B; Zimmerberg et al., 2003; Yin et al., 2016). Control animals also differed significantly from DISC1, with maternal separation and DISC1 maternally separated with ketamine injection (*p* ≤ 0.01; Figure 4B). Affective vocalizations differed to varying degrees in control animals that were maternally separated and/or treated with ketamine (for traces of USVs see also Figure 4C). On the other hand, DISC1 animals with interventions did not exhibit a reduction in calls when compared to DISC1 animals without interventions.

### Habituation to Acoustic Startle Response (ASR)

**Figure 5:**
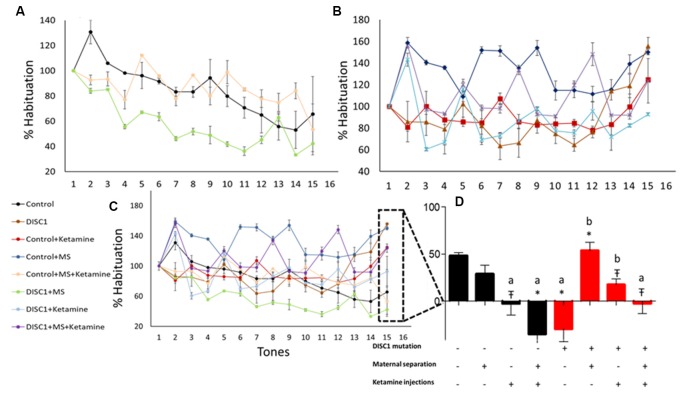
Habituation to acoustic startle response (ASR). **(A)** Groups showing habituation; control (black), control+MS (green) and DISC1+MS (pink). **(B)** Groups that did not show habituation. **(C)** Merge showing all the groups with and without habituation. **(D)** Percent habituation in different groups towards the last tone. All data presented here are presented as mean ± SEM, where a* as compared to control at *p* ≤ 0.001; a# as compared to control at *p* ≤ 0.05; a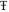 as compared to control at *p* ≤ 0.01 and *b* with above symbols shows comparison of groups with DISC1 mutation animals.

Habituation to acoustic startle response (ASR) was measured to determine sensorimotor gating and pre-attentive deficits. As previously described, the first test block measured habituation (Sultana et al., 2019). We found that maternal separation of the control pups and DISC1 pups did not affect habituation to ASR vs. control, exhibiting habituation of 65%, 53% and 42% (respectively for control, control+MS and DISC1+MS; Figures 5A,D). The magnitude of habituation was not significantly different in these animal groups. However, all other groups (see Figure 5B, control and DISC1 with ketamine, control and DISC1 with MS+ketamine, and DISC1 alone) did not show habituation but instead exhibited sensitization to the acoustic stimulus (Figures 5B,D). The animals exhibited increased startle, indicating that pharmacologically induced NMDAR hypofunction might be affecting the ability of the brain to habituate to the repeated acoustic stimulus.

### Prepulse Inhibition (PPI)

**Figure 6:**
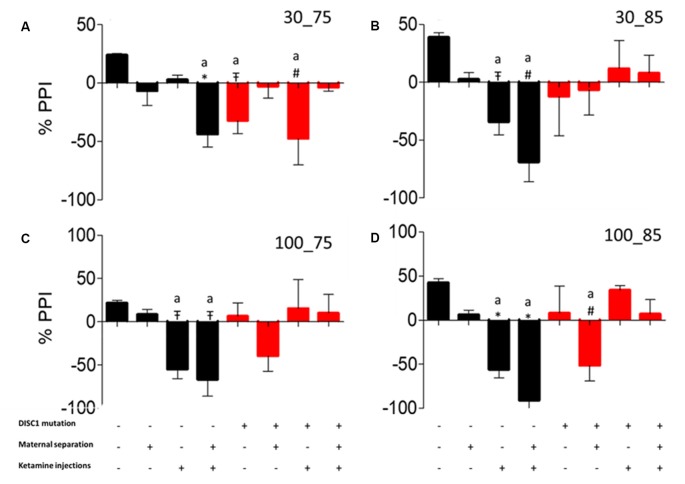
Pre-pulse Inhibition (PPI). Prepulse inhibition at different inter-stimulus interval and pulse intensity. **(A)** 30_75. **(B)** 30_85. **(C)** 100_75. **(D)** 100_85, respectively. All data presented here are presented as mean ± SEM, where a* as compared to control at *p* ≤ 0.001; a# as compared to control at *p* ≤ 0.05; a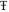 as compared to control at *p* ≤ 0.01 and *b* with above symbols shows comparison of groups with DISC1 mutation animals.

Similar to the habituation test, the PPI responses exhibited a similar pattern of inhibition to different inter-stimulus interval (ISI) and pre-pulse intensity combinations. We found that control animals showed inhibition to all trial (ISI-PP intensity) combinations (see Figures 6A–D), significantly differing from control+MS+ketamine (*p* ≤ 0.001), DISC1 (*p* ≤ 0.05) and DISC1+ketamine (*p* ≤ 0.01; at ISI of 30 ms with intensity of 75 dB depicted as 30_75 shown in Figure 6A), control+ketamine (*p* ≤ 0.05) and control+MS+ketamine [*p* ≤ 0.01; at 30 (ISI)_85 (Prepulse intensity); Figure 6B], and control vs. control+ketamine, control+MS+ketamine [*p* ≤ 0.05 at 100 ms (ISI)_75 dB (Prepulse Intensity); Figure 6C and *p* ≤ 0.001 at 100 ms (ISI)_85 dB (Prepulse Intensity) combinations; Figure 6D]. Control animals also differed from DISC1+MS [*p* ≤ 0.01 at 100 ms (ISI)_85 dB (Prepulse Intensity; Figure 6D)]. Thus, unlike habituation to acoustic startle stimulus, maternal deprivation alone in these animals did not cause aberrations in PPI (Ellenbroek and Cools, 2002). Both control and DISC1 animals with ketamine injections exhibited aberrations in PPI behavior, showing an impact of NMDAR hypofunction on sensorimotor gating mechanism of brain circuitry (Cilia et al., 2007).

## Discussion

Schizophrenia is a neuropsychiatric disorder associated with multiple genetic and environmental etiologies (Tsuang et al., 2001). Although no single gene or environmental factor is known to be completely causal (Choi et al., 2009; van de Leemput et al., 2016; Howes et al., 2017), interactions among multiple factors increase the emergence of the disorder. We focused our studies on two different genetic backgrounds, control group (C57BL/6J) which does not have a genetic predisposition to schizophrenia or schizotypic disorders, and the test group that is genetically predisposed (129S strain with C-terminal truncation of *DISC1* gene) on a behavioral test battery (Brixey et al., 1993; Krystal et al., 1994; Ellenbroek and Riva, 2003; Koike et al., 2006).

We have previously observed that this 129S: ΔDISC1 strain differs behaviorally from several other common inbred and outbred mouse strains (Sultana et al., 2019). Our prior findings indicate that the inherent DISC1 mutation in the 129SvEv mice has a penetrant effect on behavioral phenotype above various genetic backgrounds. Other mouse strains (Balb/c, CBA/J, etc.) could potentially serve as an appropriate behavioral control strain here, since they are all similar behaviorally and distinct from the 129SvEv strain, putatively as a result of the DISC1 genetic mutation. This is supported by findings that DISC1 mutations on the same background strain do not differ when compared across strains (Lee et al., 2011). Nevertheless, the comparisons employed here do add a potential caveat to our results, which must be considered in their interpretation.

DISC1 mutations affect behavior by its interactions with pathways such as PDE4, upregulation of SK2 (calcium-activated small potassium channels at the PSD; Sultana et al., 2018), and HCN (Paspalas et al., 2013). These interactions take place at the dendritic synaptic densities, where NMDARs acts as a convergence point for DISC1 and its interacting partners such as PDE4. NMDAR hypofunction alone or in combination with DISC1 mutations aggravate behavioral phenotypes, as discussed below (see also Figures 1–3).

We assessed the effects of genetic predisposition (DISC1 mutation), environmental factors (maternal separation and ketamine injections), and interactions of these factors (see Table 1). Among these factors, our results suggest that genetic factors play the predominant role in the presentation of behavioral phenotypes associated with the disease, while pharmacological intervention (ketamine injections) and maternal separation showed incremental effects, particularly on the genetically predisposed animals. Interestingly, maternal separation did not show a significant effect in terms of sociability novelty, overall activity (Figure 1), USV (Figure 4B), and habituation to acoustic startle (Figure 5) on control animals suggesting that genetic predisposition might be necessary for this stressor to contribute to disease pathology to exhibit above mentioned behavioral syndrome.

In many of the behavioral tasks, environmental interventions on the DISC1 background did not increase the severity of behavioral phenotypes. We suggest that the severity of symptoms in many of the behavioral task have a lower/upper limit. However, an interesting finding from our data is that the animals harboring the DISC1 mutation often exhibit a bimodal distribution in the expression of behavioral phenotypes, which is not found following environmental interventions. Thus, we propose that the effects of the environmental interventions may not necessarily be on the magnitude of the behavioral effects, but rather the probability that these animals may develop schizophrenia-related behavioral phenotypes.

As models to study schizophrenia, these interventions are argued to have faced, construct and/or predictive validity (Jones et al., 2011). The DISC1 mutation is known to associate with a neurological disorder in about 33.3% of the large Scottish population where the mutation is present, with members of the family exhibiting schizophrenia, bipolar and major depression disorders etc. In the present study, DISC1 mutation animals exhibited decreased sociability, novelty (Figure 1), mobility time in forced swim and tail suspension test (Figures 3, 4A). When compared with prior studies, animal models of DISC1 mutation produced using various methods, e.g., shRNA, use of chemicals, backcrossing 129S on C57BL6 background, report similar results to our findings (Jaaro-Peled, 2009; Johnstone et al., 2011; Tomoda et al., 2016). Moreover, disruption of sensorimotor gating has been observed in various models of schizophrenia including the DISC1 genetic mutation (Tomoda et al., 2017).

Additionally, NMDAR hypofunction is a key finding in human postmortem studies of schizophrenia and bolsters the glutamate dysfunction theory of schizophrenia pathogenesis (Snyder and Gao, 2013). Furthermore, it is known that DISC1 and NMDARs interact dynamically with each other (Wang and Zhu, 2014), such that DISC1 dependent changes in NMDAR synaptic responses are speculated to affect cognition in individuals with schizophrenia (Ramsey et al., 2011; Wei et al., 2014). Behaviorally, previous results from our lab also found that there is a reduced interaction of test mice in terms of sociability, social novelty, reduced spatial/working memory (Ogundele and Lee, 2018), similar to our results in ketamine-treated animals (control as well as DISC1 mutation background). We also found that ketamine injection in the DISC1 mutation animals resulted in increased hypo-frontality, leading to enhanced negative signs, such as more depressed behavior (Figures 3, 4), as indicated by decreased mobility in FST and TST as well as the reduced number of stress calls in these animals.

Genetic mutation and environmental stress affect the behavioral emergence of schizophrenia. However, when there is a combination of factors, we found that genetic background has the biggest influence as shown by reduced sociability and novelty in the DISC1 mutation background animals (Figures 1C,D), when compared with the same insults on the control background highlighted in terms of anxiety-like behavior, stress calls and habituation to ASR as well as PPI (Figures 1A, 4B, 5, 6). Environmental factors such as pharmacological interventions that cause direct NMDAR hypofunction (ketamine injections) results in similar behavioral outcomes (such as reduced sociability and mobility in FST and TST) for both control and DISC1 mutation animals, showing that NMDAR hypofunction is a convergence point for the molecular mechanism behind core symptoms of schizophrenia. On the other hand, maternal separation of pups leads to more negative symptoms, i.e., reduced mobility in FST and TST and spatial memory, but it does not affect the social recognition behavior of these animals (Figures 1C,D). All other combinations of interventions influenced the behavioral phenotype to a variable degree with DISC1+MS+ketamine animals showing more aberrant behaviors when compared with the control+MS+ketamine group, clearly indicating the effects of genetic predisposition.

Overall, our study supports the most recent theories of gene-environment interactions and their effects on the behavioral phenotype of nervous disorders, such as schizophrenia. Interactions between multiple components affect behaviors at various levels for positive (aberrant PPI, reduced habituation to acoustic startle) and negative symptoms (decreased mobility timing on FST and TST tests; Figures 3, 4), including cognitive tasks with learning and memory deficits (as shown in Y maze test; Figure 2; Ellenbroek and Cools, 2002; Powell and Miyakawa, 2006; Gómez-Sintes et al., 2014). We also emphasize the finding that, although DISC1 mutation animals with various environmental interventions did not change the severity of the behavioral profile of these animals when compared to DISC1 mutation alone, the prevalence of animals exhibiting aberrant behavioral phenotype increased due to gene-environmental interaction. We propose then that this effect could be due to environmental intervention acting as a second hit to increase the chances of disease development in genetically predisposed animals with the DISC1 mutation.

## Conclusion

These behavioral changes suggest several aberrant molecular interactions must be occurring at the cellular, subcellular and/or extracellular levels. Here, we have first attempted to assess the combination of environment and genetics in the development of behavioral phenotype. The results of our present study suggest that the DISC1 genetic mutation predominates over the environmental factors used in our study in the presentation of schizophrenia-like behavioral phenotypes. The molecular and neural factors that lead to these behaviors remain to be examined, as are any potential epigenetic changes that these stressors may bring about in healthy individuals (Roth et al., 2009).

## Data Availability Statement

The datasets generated for this study are available on request to the corresponding author.

## Ethics Statement

The animal study was reviewed and approved by Institutional Animal Care & Use Committee (IACUC) of the Louisiana State University School of Veterinary Medicine.

## Author Contributions

CL designed the study, edited and finalized the manuscript. RS designed, executed the experiments, collected data, statistically analyzed and wrote the manuscript.

## Conflict of Interest

The authors declare that the research was conducted in the absence of any commercial or financial relationships that could be construed as a potential conflict of interest.
